# Effect of probiotics on pain and ulceration in patients undergoing fixed orthodontic treatment

**DOI:** 10.34172/joddd.41128

**Published:** 2024-06-24

**Authors:** Tannaz Abdollahzadeh Baghaei, Katayoun Katebi, Mahsa Jafari Tayebpour, Mohsen Hashemi

**Affiliations:** ^1^Department of Orthodontics, Faculty of Dentistry, Tabriz University of Medical Sciences, Tabriz, Iran; ^2^Department of Oral and Maxillofacial Medicine, Faculty of Dentistry, Tabriz University of Medical Sciences, Tabriz, Iran; ^3^Faculty of Dentistry, Tabriz University of Medical Sciences, Tabriz, Iran; ^4^Department of Oral and Maxillofacial Surgery, Faculty of Dentistry, Tabriz University of Medical Sciences, Tabriz, Iran

**Keywords:** Orthodontics, Pain, Probiotics, Ulcer

## Abstract

**Background.:**

Ulcers caused by mucosal irritation of fixed orthodontic appliances remain an unsolved problem, and more research is needed to improve the pain caused by orthodontic appliances to provide more comfortable treatment for these patients. This study investigated the effect of probiotic Lactogum on pain and ulceration in patients undergoing fixed orthodontic treatment.

**Methods.:**

In this study, 64 patients over 12 years of age and candidates for fixed orthotic treatment were divided into case and control groups (n=32). The control group received orthodontic waxes, and the case group received the same orthodontic waxes and "Lactogum" probiotic lozenges from the beginning of the treatment. The number of ulcers, the amount of pain, and the location of the ulcer were recorded and compared between the two groups. An independent-sample t-test was used to compare the pain level and number of ulcers between the two groups. A significance level of 0.05 was considered for all tests. SPSS 17 was used for data analysis.

**Results.:**

The mean number of ulcers in the case group was significantly lower than the control group (*P*<0.001). The mean pain in the case group was significantly lower than in the control group (*P*<0.001). The most frequent location of ulcers was the buccal mucosa, followed by the labial mucosa.

**Conclusion.:**

Lactogum probiotic lozenges can reduce traumatic oral ulcers and pain levels in patients undergoing fixed orthodontic treatment. However, larger clinical trials are encouraged to confirm these findings.

## Introduction

 Damage to the oral mucosa during orthodontic treatment is prevalent, and the most common patient complaints are related to ulcers in the labial or buccal mucosa.^1^ Various levels of pain have been reported by approximately 95% of the patients undergoing orthodontic procedures.^2^ Ulcerations can happen due to brackets, bands, arch wires, and long unsupported sections of wire leaning on the lips.^3^ Furthermore, they can be triggered by unprovoked muscular movements of the masticatory muscles or tongue.^4^ Although these ulcers are temporary and might look minor to the dentist, they can interfere with the patient’s adherence to the treatment protocol. Currently, orthodontists seek help from auxiliary treatments to relieve the pain caused by these mucosal ulcers. One of these adjunctive treatments is giving patients wax to cover brackets or other irritating components of fixed appliances.^5^ This method is considered a symptomatic treatment because the patient uses it after irritating the mucous membrane. In addition, these waxes remain in place for a short period and need constant renewal.

 Probiotics are non-pathogenic living microbes that are used to improve the microbial balance.^6^ Probiotics are good bacteria that support a healthy immune system and digestion and provide various health benefits to the patient.^7^ The most common probiotic bacterial strains belong to *Lactobacillus *and *Bifidobacterium* genera.^8^ *Lactobacilli* usually constitute less than 1% of the total culturable microbiota in the oral cavity. Species commonly isolated from saliva samples include *L. paracasei*, *L. plantarum*, *L. rhamnosus*, and *L. salivarius*.^9,10^ The general mechanisms of probiotics can be divided into three main categories: normalization of microbiota, modulation of immune responses, and metabolic effects.^11,12^

 A study reported that *Lactobacillus brevis* reduced almost 50% of the persistence of traumatic oral lesions in patients with fixed orthodontics.^13^ Considering the significant effect of the pain and ulcers of orthodontic treatments on patients’ quality of life and the lack of definite treatment or prevention method, this study compared the effect of probiotic lozenges along with orthodontic wax on the amount of pain and ulcers in patients undergoing fixed orthodontic treatment.

## Methods

 This parallel, double-masked, randomized clinical trial was conducted at the Faculty of Dentistry, Tabriz University of Medical Sciences, Iran, from April 2022 to April 2023.

**Figure 1:**
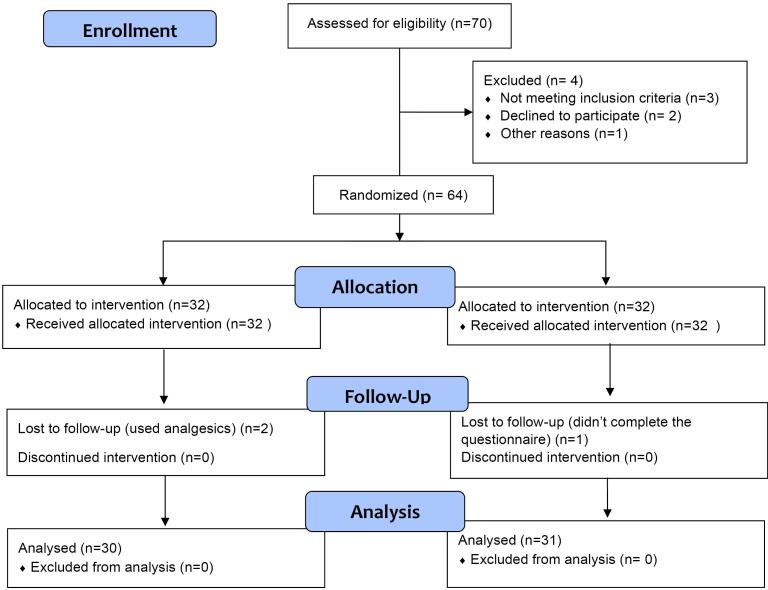
CONSORT 2010 flow diagram of study participants

 Seventy consecutive patients who attended the Department of Orthodontics and required fixed orthodontic treatment were informed about this study and were subsequently invited to participate. The CONSORT flow diagram in Figure 1 depicts the passage of patients through the trial.^14^ The selection criteria for this study were: patients aged over 12 requiring fixed orthodontic treatment; patients in good general health, without any systemic illness; patients with good oral health, in terms of dental (absence of cavities), periodontal (absence of gingivitis, active periodontal pockets or history of periodontal disease), and soft tissue (no mucositis or any bullous/erosive disease), patients who were willing and able to cooperate in all aspects of the protocol, and those who could communicate effectively and give informed written consent. Subjects were not included if any of the following criteria were present: history of hypersensitivity or allergy to the materials or drugs used in the study; the presence of auxiliary extraoral appliances that may cause additional injuries during treatment; and pregnant or lactating women and consumption of tobacco, alcohol, and any addictive substance or refusal to participate.

 Sixty-four patients were included in this study, who were randomly divided into two groups. A permuted-block randomization was performed to reach a similar female-to-male ratio in both groups. The products were coded in identical opaque containers by an operator outside the study for allocation concealment. A code for randomization was kept in an opaque envelope in a safe place and opened only at the end of the experiment. Both patients and researchers who collected the data were blinded to group assignment.

 The patients attended their scheduled appointments for bracket cementation. Metal brackets (Protec, Vancouver, Canada) were bonded on the teeth of both upper and lower arches. First, the surfaces of the teeth were washed and dried. The surface of the teeth was acid-etched for 15 seconds, washed, then dried, and BracePaste (American Orthodontics, Sheboygan, WI) adhesive was applied to the tooth surface and cured for 20 seconds. A BracePaste composite resin (American Orthodontics, Sheboygan, WI) was used in the last bonding stage. After placing it on the tooth surface, the bracket was placed on the composite and cured for 20 seconds in the incisal third using an LED device (Diadent, Seoul, Korea) at a light intensity of 1000 mW/cm^2^ for 20 seconds. Then, the 0.12” (diameter) nickel-titanium wires were placed in their correct positions. The ends of the wires were trimmed so as not to injure the mucous membrane. The hooks on the bands and brackets were trimmed if they were protruding. All the patients were provided with orthodontic wax as part of the standard procedure after cementation to prevent friction. and it was explained to them that if the components of the orthodontic device caused an ulcer in the oral tissue, they should soften the wax in their hand and place it on the part that caused the ulcer.

 The case group received a probiotic lozenge, Lactogum (Zist Takhmir, Tehran, Iran), and the control group received placebo lozenges with similar taste and packaging. The instructions were as follows: After brushing your teeth, use a lozenge once a day, give the lozenges enough time to dissolve completely in the mouth, and avoid any liquid or food intake for half an hour after application.

###  Outcomes

 The presence of traumatic oral ulcers was the primary outcome, and it was evaluated dichotomously (presence or absence). Both control and case groups received questionnaires with 28 separate columns (28 columns for 28 days) where the patients were asked to answer three questions in each row, which included:

1. The number of ulcers in the oral mucosa
2. The pain felt in the ulcers according to the rating from 0 to 10 by the visual analog scale (VAS), with 10 indicating the most severe and 0 indicating no pain.
3. Ulcer location (labial mucosa, buccal mucosa and tongue)

In this way, they wrote down all the characteristics and changes in oral ulcers that they witnessed in their mouths every day for 28 days.

###  Ethical aonsiderations

 Written informed consent was obtained from all the patients before entering the study, and the participants were assured that all the information included would be reported confidentially only for scientific purposes. They were also assured that their participation or non-participation in the study did not affect their treatment process. For the participants under 18, informed consent was obtained from their parents, and the teenager’s consent was obtained as well.

###  Statistical analyses

 Data were reported as mean ± standard deviation or percentage. An independent-sample t-test was used to compare the pain level and number of ulcers between the two groups. A significance level of 0.05 was considered for all the tests. SPSS 17 was used for data analysis.

## Results

 Of 64 patients included in the study, two participants from the case group and one from the control group were lost to follow-up (Figure 1). Finally, 61 patients over 12 years of age and candidates for fixed orthodontic treatment finished the study: 31 patients in the control groups who received placebo lozenges and 30 in the case group who received probiotic lozenges. The mean age of the participants was 19.1 ± 2.3 in the case group and 18.8 ± 3.1 in the control group.

**Figure 2:**
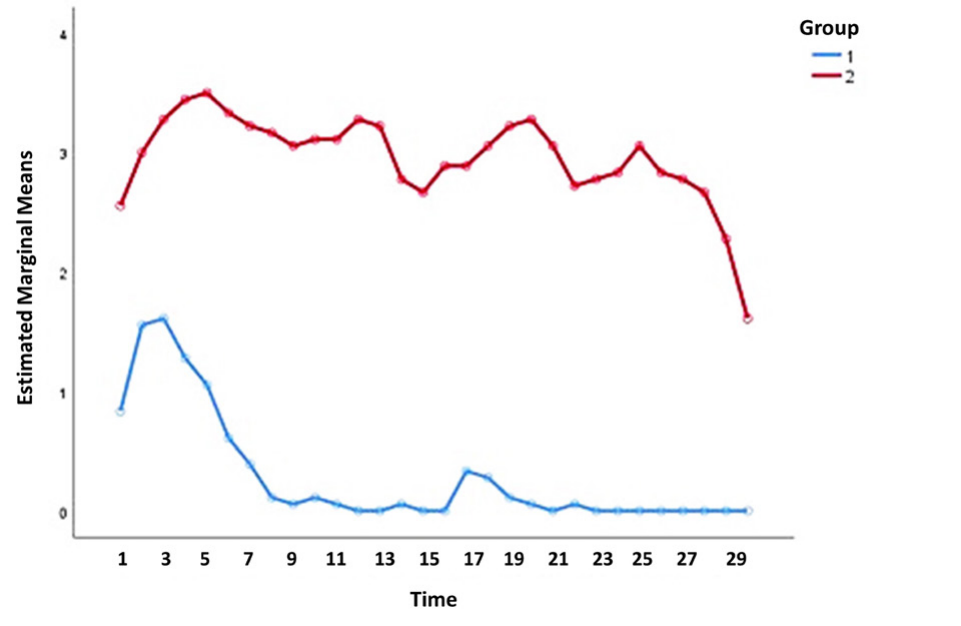
Comparison of the number of ulcers between the two groups

Figure 2 shows the trend of changes in the number of ulcers around the orthodontic device in the two groups. The mean number of ulcers in the case group was statistically lower than the control group (*P* < 0.001). Figure 2 shows that in the case group, the mean number of ulcers almost reached zero after the 8th day of treatment, while this did not happen in the control group, even on the 30th day of treatment (Table 1).

**Table 1:** Comparison of the number of ulcers in two groups

	**Groups**	**N**	**Mean**	**SD**	**P-value**	**95% Confidence interval**	
						**Lower bound**	**Upper bound**
Female	Case	15	0.3	0.1	< 0.001	0.24	0.35
	Control	15	3.0	0.1		2.94	3.05
Male	Case	15	0.2	0.1	< 0.001	0.14	0.25
	Control	16	2.9	0.3		2.75	3.04
Total	Case	30	0.3	0.2	< 0.001	0.22	0.37
	Control	31	2.9	0.2		2.83	2.97

**Figure 3:**
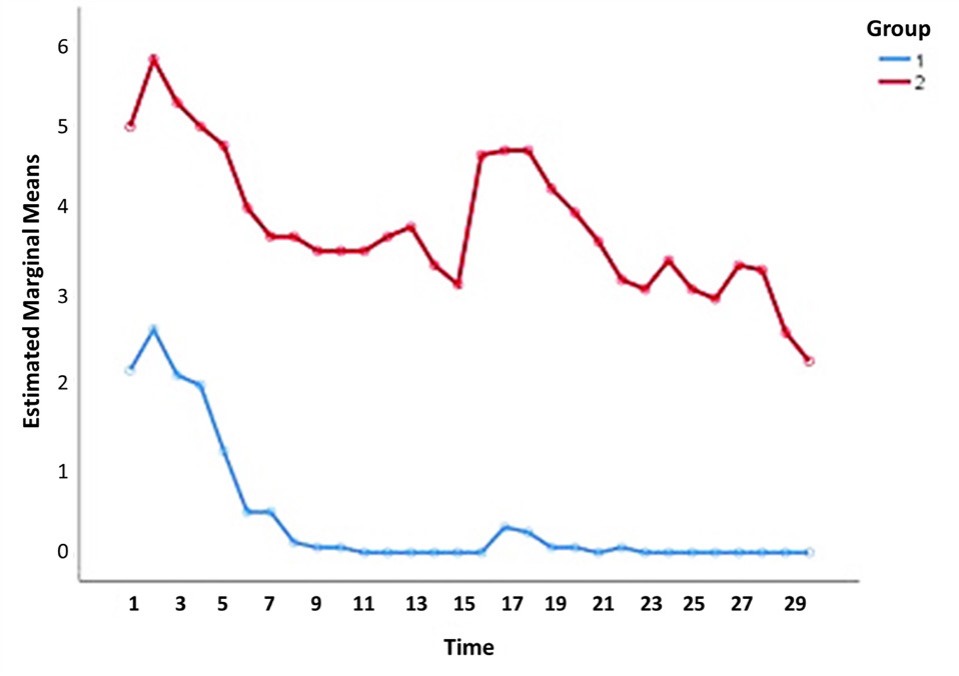
Comparison of the pain level between the two groups

Figure 3 shows the trend of pain level changes in two groups of studied patients. The mean pain in the case group was significantly lower than the control group (*P* < 0.001). Figure 3 shows that in the case group, the mean pain after the 9th day of treatment was almost zero, while this did not happen in the control group, even on the 30th day of the treatment (Table 2).

**Table 2:** Comparison of pain level in two groups

	**Groups**	**N**	**Mean**	**SD**	***P*** ** value**	**95% Confidence interval**	
						**Lower bound**	**Lower bound**
Female	Case	15	1.4	0.3	< 0.001	1.24	1.55
	Control	15	3.7	0.5		3.44	3.95
Male	Case	15	1.3	0.5	< 0.001	1.04	1.55
	Control	16	3.8	0.6		3.50	4.09
Total	Case	30	1.3	0.4	< 0.001	1.15	1.44
	Control	31	3.8	0.6		3.59	4.01

 In 15 participants (83.3%) in the case group and in all the control group patients, ulcers were present in the buccal mucosa. In 14 participants (77.8%) of the case group and 94.4% of the patients in the control group, ulcers were observed on the labial mucosa. In one participant (5.6%) of the case group and three participants (16.7%) of the control group, an ulcer was observed on the tongue.

## Discussion

 In the current study, two groups of fixed orthodontic candidates who received probiotics (case group) and those who did not (control group) were studied to investigate probiotics’ effect on pain and ulcers occurring in the oral mucosa during fixed orthodontic treatment. The mean pain in the case group compared to the control group was significantly lower than in the control group. Previous research has also shown that probiotic bacteria such as *Lactobacillus* strains present in food supplements significantly prevent the expression of inflammatory mediators by controlling the microbial balance of the oral cavity.

 Other studies used other methods to achieve this goal. In a clinical trial conducted by Kluemper et al,^15^ benzocaine-containing wax was used to reduce the pain of ulcers caused by fixed orthodontic appliances. These waxes had the property to release benzocaine gradually and exerted a soothing effect on the ulcers. The results of their study showed that the mean pain in the group that used unmedicated wax was higher than the group that used benzocaine-containing wax. Also, another clinical trial showed that the group that used aloe vera gel had significantly fewer ulcers than the group that used chlorhexidine gel.^16^

 The distribution of ulcers in both groups was similar, and they were mostly observed in the buccal and labial mucosa. The buccal mucosa is highly susceptible to ulceration because the end of the device is in the buccal mucosa, and the wire usually protrudes from the buccal tube in this area. Since there were ulcers in the mouth for a mean of 28 days in the control group and 8 days in the case group, the importance of probiotics on ulcers can be shown.

 Silva et al^13^ showed that *L. brevis* CD2 soluble tablets used in the first 21 days of orthodontic treatment reduced the length of traumatic oral lesions from 4.9 to 2.5 days. Oral pain related to traumatic lesions was also alleviated with the use of probiotics. However, there was no significant improvement in the quality of life compared to the placebo, suggesting that differences in duration and pain related to oral lesions might be clinically irrelevant.

 The signaling of toll-like receptors by the commensal microbiota plays a significant role in epithelial homeostasis, immune regulation, and protection from epithelial injuries.^17^ Probiotics can identify pattern-recognition molecules from commensal microorganisms that induce toll-like receptors and produce epithelial repair factors.^18^ It has been shown that probiotic bacteria can improve intestinal barrier function and modulate signal transduction pathways in immune and epithelial cells.^19^ Moreover, probiotics regulate the production of proinflammatory cytokines, including interleukin-1β and tumor necrosis factor-α.^20^ These possible factors could explain the mechanism of probiotics in reducing pain and ulcers in orthodontic patients. Since data on this issue is still too sparse to explain the molecular and biological mechanisms of probiotics on oral health, more investigation on the bacterial count and cytokine profile could shed light on the colonization and immunomodulation effects of the probiotic microorganisms.

 Limitations of this study are that two participants were excluded because they had taken analgesics for their pain, and the diet of patients was not controlled, so some patients in the control group might have been using probiotic yogurt. It is suggested that similar studies be conducted in patients using removable orthodontics. The optimal daily dose of probiotics has not yet been established. Also, comparisons of different probiotic strains can be useful, and it is possible that a combination of probiotic strains could be even more effective.

## Conclusion

 This study showed that Lactogum probiotics used in the first 30 days of orthodontic treatment decreased the traumatic ulcers of the mouth. Probiotics also reduced oral pain related to traumatic lesions. These findings suggest the possible effect of probiotics on reducing pain and ulcers in patients undergoing fixed orthodontic treatment. However, larger clinical trials are recommended to confirm these findings.

## Competing Interests

 The authors declare no conflicts of interest.

## Ethical Approval

 The protocol of this study was approved by the Ethics Committee (IR.TBZMED.REC.1401.589) and registered at the Iranian Registry of Clinical Trials (identifier: IRCT20230208057356N1).

## Funding

 None.

## References

[1] Alotaibi S (2023). Potential side effects of comprehensive fixed orthodontic treatment: a narrative review. Open Dent J.

[2] da Costa EO, Blagitz MN, Normando D (2020). Impact of catastrophizing on pain during orthodontic treatment. Dental Press J Orthod.

[3] Namdar P, Karkhi H, Rezaei Kalantari N, Hossein Nataj A, Namdar M, Arab S (2023). Incidence of mucosal lesions and pain during orthodontic treatment with fixed versus removable orthodontic appliances. Iran J Orthod.

[4] Aldahash F, Alshamali D, Albander W, Bakhsh R, Almadhi W, Alsenani S (2020). Oral mucosal ulceration during orthodontic treatment: the perception of patients and knowledge and attitude of the orthodontic practitioners. J Family Med Prim Care.

[5] Pasaoglu Bozkurt A, Ünlü Ö, Demirci M (2020). Comparison of microbial adhesion and biofilm formation on orthodontic wax materials; an in vitro study. J Dent Sci.

[6] Contaldo M, Lucchese A, Lajolo C, Rupe C, Di Stasio D, Romano A (2021). The oral microbiota changes in orthodontic patients and effects on oral health: an overview. J Clin Med.

[7] Wang X, Zhang P, Zhang X (2021). Probiotics regulate gut microbiota: an effective method to improve immunity. Molecules.

[8] Cizeikiene D, Jagelaviciute J (2021). Investigation of antibacterial activity and probiotic properties of strains belonging to Lactobacillus and Bifidobacterium genera for their potential application in functional food and feed products. Probiotics Antimicrob Proteins.

[9] Kim H, Fugaban JII, Holzapfel WH, Todorov SD (2022). Selection of beneficial bacterial strains with potential as oral probiotic candidates. Probiotics Antimicrob Proteins.

[10] Mann S, Park MS, Johnston TV, Ji GE, Hwang KT, Ku S (2021). Isolation, Characterization and biosafety evaluation of Lactobacillus fermentum OK with potential oral probiotic properties. Probiotics Antimicrob Proteins.

[11] Alp S, Baka ZM (2018). Effects of probiotics on salivary Streptecoccusmutansand Lactobacillus levels in orthodontic patients. Am J Orthod Dentofacial Orthop.

[12] Kaźmierczyk-Winciorek M, Nędzi-Góra M, Słotwińska SM (2021). The immunomodulating role of probiotics in the prevention and treatment of oral diseases. Cent Eur J Immunol.

[13] Silva N, Della Bona A, Cardoso M, Callegari-Jacques SM, Fornari F (2021). Lactobacillus brevis CD2 attenuates traumatic oral lesions induced by fixed orthodontic appliance: a randomized phase 2 trial. Orthod Craniofac Res.

[14] Schulz KF, Altman DG, Moher D (2010). CONSORT 2010 statement: updated guidelines for reporting parallel group randomised trials. BMJ.

[15] Kluemper GT, Hiser DG, Rayens MK, Jay MJ (2002). Efficacy of a wax containing benzocaine in the relief of oral mucosal pain caused by orthodontic appliances. Am J Orthod Dentofacial Orthop.

[16] Leiva-Cala C, Lorenzo-Pouso AI, Centenera-Centenera B, López-Palafox J, Gándara-Vila P, García-García A (2020). Clinical efficacy of an Aloe vera gel versus a 012% chlorhexidine gel in preventing traumatic ulcers in patients with fixed orthodontic appliances: a double-blind randomized clinical trial. Odontology.

[17] Semin I, Ninnemann J, Bondareva M, Gimaev I, Kruglov AA (2021). Interplay between microbiota, toll-like receptors and cytokines for the maintenance of epithelial barrier integrity. Front Med (Lausanne).

[18] Liu Q, Yu Z, Tian F, Zhao J, Zhang H, Zhai Q (2020). Surface components and metabolites of probiotics for regulation of intestinal epithelial barrier. Microb Cell Fact.

[19] Rose EC, Odle J, Blikslager AT, Ziegler AL (2021). Probiotics, prebiotics and epithelial tight junctions: a promising approach to modulate intestinal barrier function. Int J Mol Sci.

[20] Faghfouri AH, Gol Mohammad Pour Afrakoti L, Kavyani Z, Sadeghi Nogourani Z, Musazadeh V, Jafarlou M (2023). The role of probiotic supplementation in inflammatory biomarkers in adults: an umbrella meta-analysis of randomized controlled trials. Inflammopharmacology.

